# Klatskin Tumor: A Survival Analysis According to Tumor Characteristics and Inflammatory Ratios

**DOI:** 10.3390/medicina58121788

**Published:** 2022-12-05

**Authors:** Vlad-Ionuţ Nechita, Emil Moiş, Luminiţa Furcea, Mihaela-Ancuţa Nechita, Florin Graur

**Affiliations:** 1 Department of Medical Informatics and Biostatistics, “Iuliu Hațieganu” University of Medicine and Pharmacy, Louis Pasteur Street, No. 6, 400349 Cluj-Napoca, Romania; 2 3rd Department of Surgery, “Octavian Fodor” Regional Institute of Gastroenterology and Hepatology Cluj-Napoca, Croitorilor Street, No. 19-21, 400162 Cluj-Napoca, Romania; 3 3rd Department of Surgery, “Iuliu Hațieganu” University of Medicine and Pharmacy, Croitorilor Street, No. 19-21, 400162 Cluj-Napoca, Romania; 4 “Ion Chiricuță” Oncology Institute, Republicii Street, No. 34-36, 400015 Cluj-Napoca, Romania

**Keywords:** Klatskin tumor, inflammatory ratios, resectability, survival

## Abstract

*Background and Objectives*: The aim was to evaluate the association of inflammatory biomarkers with resectability and overall survival in hilar cholangiocarcinoma. *Materials and Methods*: We conducted a retrospective cohort study over 72 consecutive surgical cases of Klatskin tumor over an 11-year period. The sample was divided into two groups: 42 surgical resection cases and 30 unresectable tumors. Values of inflammatory ratios were compared according to the resectability. Log-rank test, univariate, and multivariate Cox proportional hazards models were used to evaluate the overall survival. *Results*: Subjects were between 42–87 years old (average age of 64.91 ± 9.15 years). According to the procedure: 58.33% benefited from resection (with a 30.95% R0 resection rate) and 41.66% had palliative surgery. Elevated NLR (neutrophil to lymphocyte ratio), PLR (platelet to lymphocyte ratio), and SII (systemic immune-inflammation index), and lower LMR (lymphocyte to monocyte ratio) at admission were associated with unresectable tumors (*p* < 0.01). For the multivariate Cox proportional hazard models, increased absolute values of NLR, PLR, and SII were associated with lower survival; no differences were observed for LMR absolute value. The cut-off value of NLR ≥ 6 was associated with lower survival. The median survival time for all subjects was 442 days, with 774 days for the resection group and 147 days for the group with palliative surgery. *Conclusions*: In hilar cholangiocarcinoma, inflammatory ratios are associated with tumor resectability. Tumor excision conferred an important advantage in survival. Elevated NLR, PLR, and SII values at admission significantly increased the hazard ratio. LMR had no influence on survival.

## 1. Introduction

Hilar cholangiocarcinoma, also called Klatskin tumor, is a malignant development of the cholangiocytes (epithelial cells of the biliary tract) in the first region of the extrahepatic bile duct, above the confluence with the cystic duct and below the second-order bifurcation bile ducts. Intrahepatic cholangiocarcinoma represents about 10% of all cholangiocarcinoma, and extrahepatic cholangiocarcinoma about 90%, with 50 to 60% of them having a hilar localization [1,2]. Tumor-free margins are difficult to obtain in Klatskin tumor cases. Tae Yoo et al., in a 12-year period study on 117 patients with Klatskin tumor who underwent curative intent surgery, showed that only 103 patients benefited of resection (88.3% resectability) and 33% of them had a positive resection margin after morphopathology examination. [3]. According to Capobianco et al., resectability ranges from 32% to 80%. Even if about 60% of the patients are eligible for the surgical procedure, about 20–30% of the patients will have a positive margin resection R1 [4,5]. Median survival without treatment in advanced stages, on condition of a natural evolution of the disease, is considered three months [6].

In a study on 95 patients who underwent laparotomy, with a resectability rate of 63%, Ruys et al. found a median survival of 37 months (43% five year-survival rate) for resected patients, and a median survival of 13 months for unresectable patients. In cases of locally advanced disease, median survival was better (13 months) than in metastasis cases (for liver metastasis, median survival was 3 months, and for other localizations, median survival was 5 months) [7].

Beckurts et al. compared the survival of patients who underwent surgical resection with the palliative treatment for Klatskin tumors on a sample of 45 subjects. A percentage of 68.9% (31 patients) benefited from surgical intervention but only 54.8% (17/31 patients) benefited from R0 free margins resection. Among patients with resection (no matter the status of the margins), the one-year survival rate was 75.4% with a median survival of 729 days; for the group with palliative treatment, the survival rate was 30.8% with a median survival of 153 days [8].

Neutrophils increase in the inflammatory process, being the first line of defense activated by metabolites derived from arachidonic acid. The malignant process induces a pro-inflammatory status. According to some studies, neutrophils are involved in the proliferation and metastasis of neoplasia, increasing the survival of cancer cells via interleukin 1, interleukin 6, and vascular endothelial growth factor (VEGF). Neutrophils are a physiologic circulating carrier of VEGF. Doses of VEGF contained in neutrophils increase in case of malignancy [9]. Tumor necrosis factor alpha (TNF alpha) can activate the release of VEGF from neutrophils and, in this way, increase the vascular permeability and supply to malignant tissue promoting tumor growth and metastasis [10,11]. On the other hand, lymphocytes are the protective part of the inflammation process. Stress induces an increase in cortisol levels that determine lymphopenia in case of inflammation. In malignant disease, lymphocytes infiltrate the tumor and determine apoptosis through cytotoxic factors released by CD8 cells [12]. Ratios like Neutrophil to lymphocyte ratio (NLR), platelet to lymphocyte ratio (PLR), lymphocyte to monocyte ratio (LMR), and SII (Systemic immune-inflammation index) are considered inflammatory biomarkers. They were studied as prognostic factors in several neoplasia such as gastroesophageal cancer, hepatocellular carcinoma, colorectal cancer, pulmonary neoplasia, ovarian cancer, breast cancer, prostate cancer, and nasopharyngeal cancer. In the case of NLR, a higher HR (hazard ratio) was observed for anal canal neoplasia (HR = 4.9) and mesothelioma (HR = 4.5) [13,14,15,16,17,18,19]. Inflammatory biomarkers increase vascular permeability, so the malignant cells can infiltrate via blood and lymphatic vessels and this leads to tumor progression. Increased inflammatory biomarkers are usually associated with poor prognosis [20].

Our aim was to evaluate the survival of Klatskin tumor patients with indication for surgery regarding the inflammatory biomarkers (NLR, PLR, LMR, SII) at admission. On the other hand, we compared the values for inflammatory ratios between the patients that are suitable for resection vs. the patients that received only palliative surgical treatment (intraoperative, they had unresectable tumors).

## 2. Materials and Methods

### 2.1. Study Design

We developed a cohort study with retrospective data collection considering Klatskin tumor patients with surgical resection or palliative surgical procedures during hospitalization in the Surgery Department of Regional Institute of Gastroenterology and Hepatology “Prof. Dr. Octavian Fodor” from Cluj-Napoca, Romania. All medical charts available from January 2011 to December 2021 were reviewed.

### 2.2. Participants

For this study were enrolled patients with confirmed Klatskin tumors with a surgical intervention (resection or palliative procedure for advanced cases). Patients without surgery, with high surgical and anesthesia risk, or with other invasive palliative interventions like endoscopic stenting or percutaneous ultrasound-guided drainage, as well as patients with only medical or oncologic treatments, or who were transferred to another service for the intervention, were excluded.

The diagnosis was assumed according to the golden standard: histopathology result. Biopsy results, operative protocol information, computer tomography, magnetic resonance, and ultrasound examinations were considered for diagnosis and staging of unresectable subjects.

### 2.3. Variables, Data Collection, and Follow-Up

Variables like age, sex, and urban setting were collected to demographically describe the sample. Tumor invasion (T), metastasis (M), lymph node invasion (N), TNM stage, and Bismuth class were used to describe the tumor status. The type of procedure and survival time were also analyzed. Overall survival was considered as the primary endpoint and represented the interval between surgery and date of death. Information about survival was obtained from population records in Cluj-Napoca up to 31 August 2022.

Laboratory data were collected from the first results available at admission into our service before other interventions or procedures. The inflammatory scores like NLR (neutrophil to lymphocyte ratio), LMR (lymphocyte to monocyte ratio), PLR (platelets to lymphocyte ratio), and SII (systemic immune-inflammation index) were computed using the absolute number of neutrophils (10^3^/μL), lymphocytes (10^3^/μL), platelets (10^3^/μL), and monocytes (10^3^/μL), divided to obtain the specific ratio. For SII the following formula was used: (platelets x neutrophils)/lymphocyte absolute values.

For blood counts, samples were collected on EDTA (ethylenediaminetetraacetic acid). Laboratory determinations were made on MINDRAY BC-6800 Hematology Analyzer (MINDRAY Headquarters, Shenzhen, 518057 P. R. China, SN SH-25000300). Vacutainer with sodium citrate was used for biochemistry, and the samples were worked on Konelab PRIME 60 ISE Type 983 (Thermo Fisher Scientific, Vantaa, Finland, SN 23040). A maximum 2 h delay from blood collection moment was accepted for analysis.

### 2.4. Statistical Analysis

Variables were collected retrospectively from the hospital database. Qualitative variables were presented as counts and percentages; quantitative data were presented as averages and standard deviations in the case of normal distribution, and median with interquartile ranges for skewed data. Survival data was compared using the Log rank test and was plotted using Kaplan–Meier curves. Next, Cox proportional hazard regressions were used. Univariate models were built with NLR, PLR, LMR, and SII, as continuous or as binary data (dichotomized with medians and cut-off values approximated from those found in the literature). Multivariate models were built for the variables of interest, adjusting for age, procedure, tumor stage (T), and metastasis (M) at the presentation for all the subjects. The subjects with resection and complete TNM stadialisation were also adjusted for TNM stage and resection margin. For all the models, we presented the hazard ratio, the 95% confidence interval, and the *p*-value. For all statistical tests used, the significance level of the two-tailed *p*-value was 0.05. All statistical analyses were done with the R environment for statistical computing and graphics (R Foundation for Statistical Computing, Vienna, Austria), version 3.6.1 [21]. Regarding the test hypothesis, we verified if survival was statistically significant different according NLR, PLR, LMR, and SII at admission, and if it there a significant difference in these ratios between patients who were eligible for resection versus the subjects discovered at an advanced stage where the intervention was limited to palliative surgery.

### 2.5. Ethical Statement

The approval of “Prof. Dr. Octavian Fodor” Regional Institute of Gastroenterology and Hepatology Ethical Committee (number 2480/23 February 2022) and Iuliu Hațieganu Ethical Committee (number 265/30 June 2021) were received for this study.

## 3. Results

**Figure 1:**
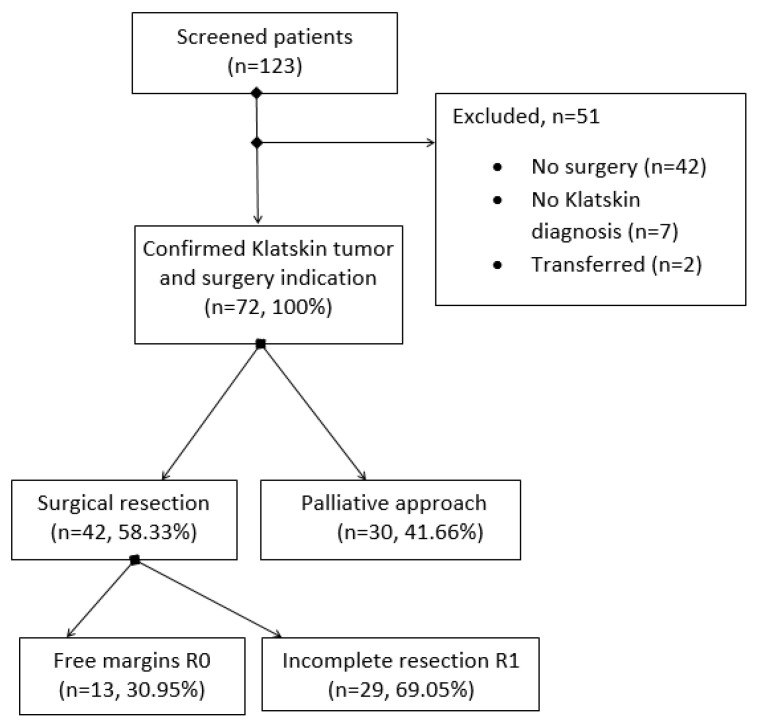
Flowchart with patients’ enrollment.

Seventy-two patients with Klatskin tumor and recommendation for surgical intervention who were presented to our surgery department during a period of 11 years (January 2011–December 2021) were considered for the evaluation. Thirty patients (41.66%) had a surgical approach without resection of the tumor mass, and forty-two patients (58.33%) had a surgical resection. A flowchart with the patients’ distribution is presented in Figure 1. 

The average age for the subjects in the study was 64.91 ± 9.15 (mean ± standard deviation) years, with values between 42 and 87 years, and a male-to-female ratio of 1.88. Other characteristics of the sample, including the inflammatory ratios, are presented in Table 1.

The major symptoms at the presentation were jaundice (76.39%) and pain (43.06%). Regarding the laboratory findings, 34.72% of the subjects presented anemia (Hb < 12 mg/dL), 84.72% had a total bilirubin > 1.2 mg/dL, and, for 44.44%, the total bilirubin was >10 mg/dL. Liver enzymes were increased (>45 U/L) for 83.33% of the subjects, alkaline phosphatase was higher than 150 (U/L) for the entire sample, and GGT (gamma-glutamyl transpeptidase) was higher than 50 U/L for 98.61% of patients.

Only about a third of the patients were classified as Bismuth I or II 26/72 (36.11%); the rest of them were Bismuth III or IV 46/72 (63.89%). Regarding the tumor stage, vascular invasion was found for 37.5% of the subjects, invasion in the nearby organs in 34.72%, and 11.11% of the subjects presented invasion in more than one organ. Twelve patients (16.67%) were discovered with metastasis, and two of them (2.78%) had metastasis in two or more organs. More characteristics regarding the tumor are presented in Table 1. Resectability rate was 58.33% with a rate of free margin resection R0 of 30.95%.

At admission, a significantly higher NLR (*p* < 0.001—Mann Whitney test), PLR (*p* = 0.005—Mann Whitney test), and SII (*p* = 0.001—Mann Whitney test), and a lower LMR (*p* < 0.001—Mann Whitney test) were observed for patients with unresectable Klatskin tumor (Table 1). 

We assessed the relationship of inflammatory biomarkers with overall survival for patients with Klatskin tumors (Table 2). In the univariate analysis, we found that higher tumor stage (T, *p* < 0.001), presence of metastasis (M, *p* < 0.001), and palliative surgical procedure (*p* < 0.001) were associated with poor survival; age (and age over 65 years) was not associated with survival (*p* = 0.72 and *p* = 0.8, respectively). 

Between inflammatory ratios, only the absolute value of LMR and a NLR higher than three at the presentation were not significantly associated with poor survival in the univariate analysis. In the multivariate analysis, only the NLR, PLR, and SII absolute values at admission remain statistically significant. An NLR higher than the median and higher than the cut-off value of 6 was significantly associated with poor survival. The other cut-off values did not make a difference in survival (Table 2). Considering only subjects with curative-intent surgical resection and adjusting over TNM stage, positive resection margin, and age ≥ 65 years, no significant differences were observed for inflammatory ratios. 

The difference in survival between the surgical resection group with a median survival time of 774 days (95% CI 563–1716) and the palliative group with 147 days (95% CI 91–301) median survival time was statistically significant (*p* < 0.001—Log Rank test). The median survival time for R1 resection was 567 days (95% CI 444–1335) vs. 2147 days (95% CI 1161—NA) for R0 resection (*p* < 0.001—Log Rank test). According to the TNM stage, the differences in survival were significant (*p* < 0.001—Log-rank test), with a higher median survival time for stage I of 1420 days (95% CI 695—NA); stage II, 1161 days (95% CI 641—NA); stage III, 514 days (95% CI 240—NA); and stage IV, 115 days (95% CI 50—NA). Subjects with metastasis have a significantly lower (*p* < 0.001—Log Rank test) median survival time of 112 days (95% CI 50—NA) vs. 563 days (95% CI 392—1161) for non–metastatic tumors. Considering local tumor invasion, the differences were significant (*p* < 0.001—Log Rank test), with a median survival time of 1429 days (95% CI 695—NA) for T1, 1161 days (95% CI 592—NA) for T2, 466 days (95% CI 277—NA) for T3, and 152 days (95% CI 91–273) for T4. No survival differences were observed regarding gender (*p* = 0.2—Log Rank test), urban setting (*p* = 0.6—Log Rank test), Bismuth type (*p* = 0.2—Log Rank test), or tumor grading (*p* = 0.1—Log Rank test). First-year survival rate for patients with surgical resection was better 73.53% (95% CI 61.26–88.3%) in comparison to those with palliative surgery 23.3% (95% CI 12.2–44.6%). Regarding the 42 subjects with resection, significant difference (*p* = 0.01—Log Rank test) was observed between R0 median survival time 2147 days (95% CI 1161—NA) and R1 567 days (95% CI 444–1335). 

**Figure 2:**
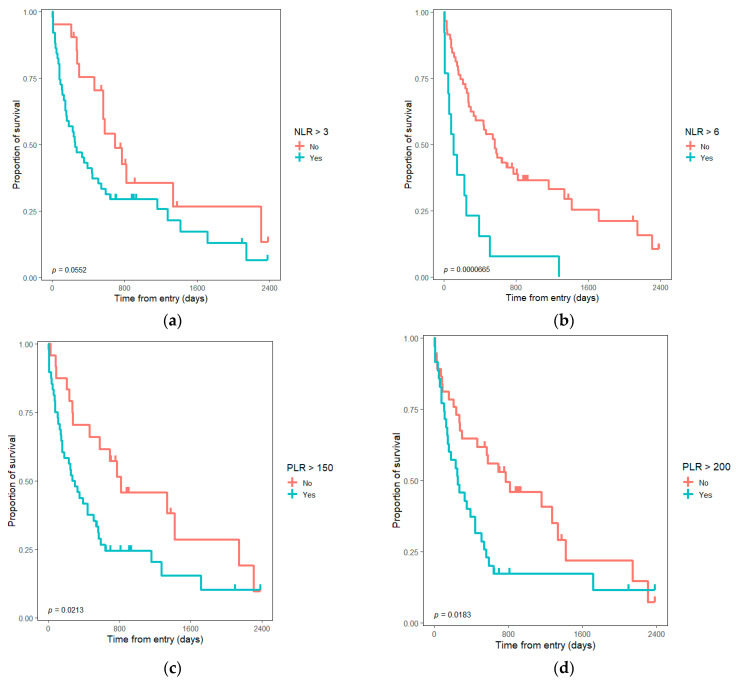

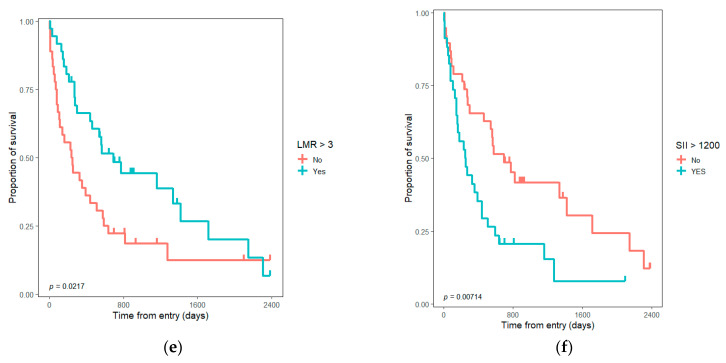
Kaplan–Meyer graph with survival differences for Klatskin tumors according to inflammatory ratios cut-off values: (**a**) survival considering NLR > 3; (**b**) survival considering NLR > 6; (**c**) survival considering PLR > 150; (**d**) survival considering PLR > 200; (**e**) survival considering LMR > 3; (**f**) survival considering SII > 1200. NLR—neutrophil to lymphocyte ratio; PLR—platelet to lymphocyte ratio; LMR—lymphocyte to monocyte ratio; SII—systemic immune-inflammation index.

**Figure 3:**
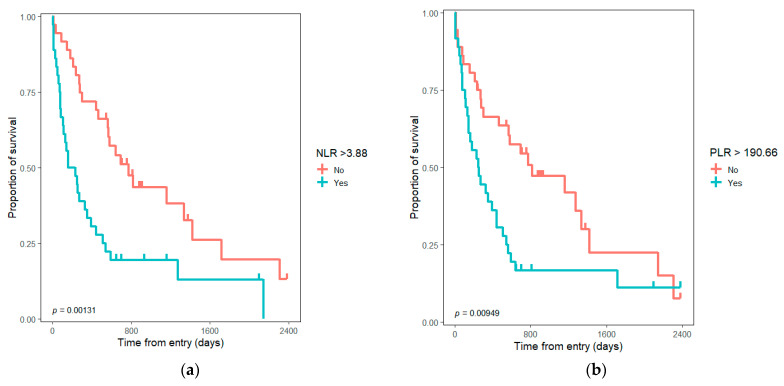

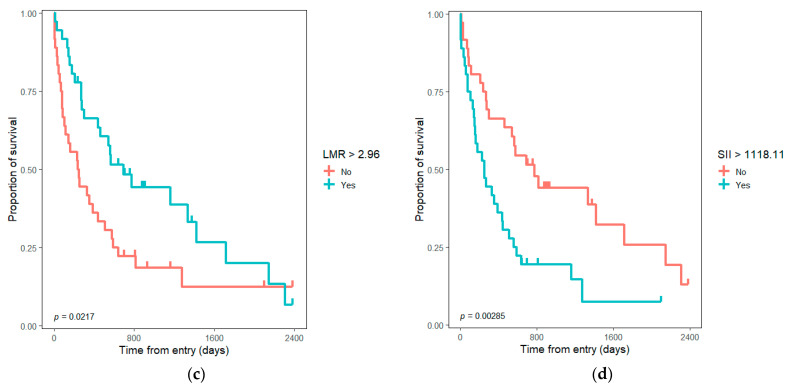
Kaplan–Meyer graph with survival differences for Klatskin tumors considering median value for inflammatory ratios: (**a**) survival considering NLR > median value; (**b**) survival considering PLR > median value; (**c**) survival considering LMR > median value; (**d**) survival considering SII > median value. NLR—neutrophil to lymphocyte ratio; PLR—platelet to lymphocyte ratio; LMR—lymphocyte to monocyte ratio; SII—systemic immune-inflammation index.

The differences in survival considering the cut-off values and the sample median values for inflammatory ratios are presented in Figure 2 and Figure 3.

## 4. Discussion

A significant difference in survival of Klatskin tumor subjects was observed regarding their inflammatory status at the presentation; furthermore, the differences at admission were significant between resectable and unresectable tumor groups. This study brings evidence that a patient’s inflammatory status can predict resectability and survival in hilar cholangiocarcinoma.

For Klatskin tumor patients, the R0 resection is the most desired treatment possibility, but with technical difficulties because of important anatomic structures nearby and the infiltrative character of the tumor, R0 is hard to reach, and resectability is difficult to predict before intervention. According to some studies, free margins can be achieved in 52–67% of the cases [3,8,22]. In our case, the rate of R0 resection was poor at only 30% (see Figure 1), maybe because many patients presented too late at the hospital. The R0 resection group was not considered independently in the analysis because of the low number of subjects (*n* = 12). Resectability rate is also debatable in the literature as Matull et al. found 28% of the patients who presented in a tertiary center like ours as eligible for surgery, and only 38% of them benefited from R0 resection [23]. There are some studies that compare R0 with R1 resection on long-term survival and find no statistically significant difference [22,24,25]. Seyama et al. found a significant difference in survival between R0 and R1 resection only if R0 margin is over 5 mm from the tumor; the R0 with a narrow margin (<5 mm) had a comparable survival with R1 resection [24].

There are also some other studies in the literature that found R1 resection to be associated with a poor survival rate (21 months), no better than cases with laparotomy, exploration, and without resection because of a locally advanced tumor (16 months); only the R0 resection (median survival 42 months) gave a significant difference (*p* = 0.0075) [5]. The sample size in our case was comparable with other studies regarding the follow-up period; Klatskin tumor is known to be a rare pathology [4,26,27]. Median survival in our palliative group of about 5 months (147 days) was comparable with the data from the literature (153 days) [8]. A significantly higher median survival was found for the patients with surgical resection, as expected, at about 25 months (774 days). Ruys et al. reported a higher median survival time in both situations: resectable (37 months) and unresectable cases (13 months) [7]. A drawback in our study was that adjuvant treatment could not be investigated properly and factored into the analysis; however, in a similar study, the adjuvant therapy group did not grant a better survival [28]. Regarding Bismuth class, Weber et al., on 76 unresectable patients, found no significant difference in survival [6]. The differences were not significant also in our study. The individual groups had too small number of subjects to consider Bismuth classes separately (see Figure 1); for further research, a larger sample size is recommended. Lymph node involvement [8,24,25,29] and tumor differentiation [30] were also reported to influence recurrence-free survival. In our cases, detailed histopathology results were available only for cases with tumor resection (*n* = 42); for them, N stage was considered in the Cox regression as part of the TNM stage for adjustment. Tumor grading did not make a difference in survival. To decide the TNM stage for Klatskin tumor according to histopathology results, we used the TNM staging classification of the American Joint Committee on Cancer (AJCC) 2018, 8th edition [31]. In this new classification, local tumor invasion T4 is considered as stage IIIB in contrast to previous classifications, where it was in stage IVA. Therefore, in the AJCC 8th edition, the lymphatic invasion N2 or presence of metastasis (M1) can bring the patient to stage IV [32]. The average age of the participants was comparable (65 years, see Table 1) with that described in the literature, being represented especially by the 6th and 7th decade [1,33]. Even if some studies describe this tumor as also highly prevalent in the octogenarian population, only two patients over 80 years were present in our sample; it is appropriate to mention that combined life expectancy in Romania is considered 76.5 years [34]. For adjustment in the Cox regression multivariate model, we also considered the age dichotomized as lower or higher than the average age for our sample, about 65 years. The higher prevalence in the male population for Klatskin tumors [1] was also consistent in our study (see Table 1). Based on these findings, we can assume that we have an appropriate demographical representation in our sample.

The inflammatory ratios are associated with poor prognosis and limited possibilities in a large variety of cancers [12,13,14,15,16,17,18,19,20]. Klatskin tumor is a rare malady [4,26,27], and even though there was an approach towards inflammatory-based prognostic scores in the literature for some individual biomarkers, our study tried to evaluate them together in a single study and to update the results for the Romanian population. Based on our research, for Romania, only two studies evaluate general survival and a third evaluates the prognostic value of inflammatory ratios for hilar cholangiocarcinoma [35]. Regarding the inflammatory biomarkers, higher NLR, PLR, and SII values, as well as lower LMR, were associated with unresectable tumors and poor prognosis. Neutrophils as natural carriers of VEGF can explain the phenomenon of increasing tumor vascularization and development [9,10,11] associated with a more advanced and aggressive tumor. Lymphocytes are involved in the cytotoxic anti-tumoral activity; a decrease in lymphocyte number can be related to a low antitumor immune response [36]. Considering these facts, a higher NLR leads to an inferior prognosis. Kitano et al. suggested the superiority of high PLR in comparison with high NLR as a prognostic factor for extrahepatic cholangiocarcinoma [29]. In their study, to the contrary, NLR did not reach the level of significance to be a prognostic value for overall survival (HR = 1.19, *p* = 0.52), but higher PLR was significantly associated with poor overall survival (HR = 1.98, *p* = 0.008). They considered PLR to be a superior and more accurate indicator for systemic inflammation in extrahepatic cholangiocarcinoma. Both markers were validated in our study (see Table 2, Figure 2 and Figure 3). Some other studies that compare PLR and NLR find NLR as a more accurate prognostic factor in neoplasia cases (colorectal and gastric cancer) [37,38]. In the univariate model, the inflammatory ratios proved significantly associated with survival for all the cut-offs and absolute values, except the LMR absolute value and NLR ≥ 3 (Table 2). The adjustments were made regarding age, the resection possibility, and tumor characteristics, such as local invasion (T), and distal invasion (M). As cut-off values, we considered the median and some values adapted from the literature for each ratio. Tan et al. evaluated, in a meta-analysis, the preoperative NLR as a prognostic factor in cholangiocarcinoma [12]. High NLR was associated with poor survival with a combined HR of 1.449 (95% CI: 1.296–1.619, *p* < 0.001); furthermore, a cut-off value of NLR ≥ 4 (HR = 1.724, 95% CI: 1.215–2.233) was described as associated with poor prognosis. For patients with resected intrahepatic cholangiocarcinoma, Zhang et al. appreciated the high NLR ≥ 2.49; HR = 1.504 (1.108–2.043) and low LMR ≤ 4.45; HR = 0.636 (0.461–0.878) as being associated with poor prognosis (*p* < 0.01) [39]. Patients with intrahepatic cholangiocarcinoma with PLR > 148 had a significantly lower 5-year overall survival (14.5% vs. 26.2%); HR = 2.06 (95% CI 1.13–3.74), *p* = 0.003 [40]. Preoperative NLR > 2.75 (OR = 3.74, 95% CI 1.09–12.83) and PLR > 172.25 (OR = 7.86, 95% CI 2.25–27.43) can predict more advanced tumor stage in hilar cholangiocarcinoma [41]. Hu et al. suggested that PLR ≥ 150 and NLR ≥ 3 were associated with the unresectable tumor for patients with hilar cholangiocarcinoma [42]. Bittoni et al. suggested a SII cut-off value of 1200 for subjects with advanced pancreatic ductal adenocarcinoma. A higher SII value is significantly (*p* = 0.0001) associated with a shorter survival HR = 2.04 (95% CI: 1.59–4.19), with a median survival of 4.8 months for SII ≥ 1200 vs. 10.1 months for SII < 1200. The value was close to the median value for our sample and used as a cut-off value, proving the same association. According to data in the literature, we can consider both preoperative and postoperative inflammatory ratios to be relevant for the prognosis. For our studied cohort, only the preoperative results were homogenously available for the eligible subjects. In addition, we considered the possible impact of the invasivity of the surgical procedure, medication, and anesthesia on the early postoperative values. In our opinion, the inflammatory ratios should not be considered alone, but in association with other tumor-related factors to appreciate the prognostic.

Our findings are consistent with the literature; higher NLR, PLR, and SII values were associated with poor overall survival in univariate analysis for both absolute and cut-off values. LMR absolute value does not validate, only the cut-off value 3 (Table 2). In the multivariate analysis, only NLR, PLR, SII absolute value, NLR ≥ 6, and NLR > median value were significantly associated with poor survival. Our results are consistent with other studies that demonstrate higher inflammatory ratios as indicators for poor survival in cholangiocarcinoma [43,44]. When we considered only the 42 subjects with resection in the multivariate analysis, the inflammatory ratios proved of no significance; a trend was observed only for the LMR absolute value (*p* = 0.055), and this group was more uniform according to the stadialisation without the unresectable cases (30 patients).

The prognostic value of inflammatory ratios was investigated for the entire sample at the presentation, i.e., before the patient was assessed to a procedure (resection or palliative treatment) and before the patient received medication, as the first laboratory results available. A possible limit in this evaluation was that associations with inflammatory diseases or anti-inflammatory drugs, other chronic treatment, and history of medication were not taken into consideration. Conditions that can influence the inflammation status of the patient might also have influence over the inflammatory ratios. Another important inflammatory biomarker, the CRP (C—reactive protein), was not homogeneously available for the retrospective evaluation, but the accessible data is presented in Table 1. A further prospective and well-documented study (in addition to a questionnaire regarding chronic disease and medication) is recommended, on a larger sample if possible. Another limitation was the single-center data collection that might limit the generalizability. Detailed pathology reports were not available for unresected cases (*n* = 30) for the additional consideration of lymph node involvement (N) and other tumor characteristics in order to assess the TNM stage properly; twelve patients without resection were classified as stage IV due to the presence of metastasis.

According to our knowledge, this is the first study in Romania to investigate the associations between inflammatory biomarkers, the procedure, and the survival rate for surgical cases of Klatskin tumors admitted to a regional center. This study adds more necessary evidence to a topic where the incidence of the disease is very low. Moreover, the multivariate analysis limits the confounding bias for the important prognostic variables used in the models.

## 5. Conclusions

Higher inflammatory ratios at the moment of patient presentation in the surgery department for Klatskin tumor are related to limited surgical possibilities and poor overall survival. Elevated NLR, PLR, and SII values at the presentation significantly increase the hazard ratio for hilar cholangiocarcinoma. Other inflammatory biomarkers such as LMR do not appear to influence survival rate.

## Figures and Tables

**Table 1:** Main characteristics of the subjects and inflammatory ratios regarding the procedure.

	All Subjects (*n* = 72)	Tumor Resection (*n* = 42)	Unresectable Cases (*n* = 30)
Age (years) ^a^	64.91 ± 9.15	65.54 ± 8.87	64.03 ± 9.62
Male gender ^b^	47 (65.27%)	28 (66.66%)	19 (63.33%)
Urban setting ^b^	39 (54.16%)	26 (61.90%)	13 (43.33%)
Bismuth class ^b^			
I	16 (22.22%)	9 (21.42%)	7 (23.33%)
II	10 (13.88%)	7 (16.66%)	3 (10%)
III	25 (34.72%)	17 (40.47%)	8 (26.66%)
IV	21 (29.16%)	9 (21.42%)	12 (40%)
Tumor stage (T) ^b^			
I	7 (9.72%)	7 (16.6%)	na
II	24 (33.33%)	24 (57.14%)	na
III	11 (15.27%)	8 (19.04%)	3 (10%)
IV	30 (41.66%)	3 (7.14%)	27 (90%)
Node stage (N) ^c^			
0	28 (66.66%)	28 (66.66%)	na
1 (1–3 regional lymph nodes)	13 (31.66%)	13 (30.95%)	na
2 (≥4 regional lymph nodes)	1 (2.38%)	1 (2.38%)	na
Metastasis (M) ^b^	12 (16.66%)	0	12 (40%)
TNM ^d^			
I	7/54 (12.96%)	7/42 (16.66%)	na
II	16/54 (29.62%)	16/42 (38.09%)	na
III	18/54 (33.33%)	18/42 (42.85%)	na
IV	13/54 (24.072%)	1/42 (2.38%)	na
Event (death) ^b^	55 (76.38%)	26 (61.9%)	26 (86.66%)
Negative resection margin (R0) ^b^	13 (18.05%)	13 (30.95%)	na
Median survival time (days)	442 (273–641)	774 (563–1716)	147 (91–301)
NLR at admission	3.88 (2.86–5.18)	3.26 (2.56–4.19)	4.84 (3.92–7.10)
PLR at admission	190.66 (133.15–317.68)	152.52 (124.81–249.96)	238.15 (182.92–352.21)
LMR at admission	2.96 (1.91–4.18)	3.48 (2.52–4.38)	1.92 (1.5–3.03)
SII at admission	1118.11 (717.94–1973.42)	850.55 (579.75–1331.35)	1673.64 (927.21–2489.5)
WBC at admission (10^3^/μL)	8.49 (6.84–10.67)	7.82 (6.03–9.93)	8.9 (7.41–11.66)
CRP at admission (mg/dL)	0.62 (0.4–2.44)	0.64 (0.39–2.9)	0.57 (0.44–1.55)
NLR > median	36 (50%)	13 (30.95%)	23 (76.66%)
PLR > median	35 (50%)	14 (33.33%)	22 (73.33%)
LMR > median	35 (50%)	27 (64.28%)	9 (30%)
SII > median	35 (50%)	15(35.71%)	21 (70%)
NLR > 3	51 (70.83)	25 (59.52%)	26 (86.66%)
NLR > 6	13 (18.05%)	3 (7.14%)	10 (33.33%)
PLR > 150	48 (66.66%)	23 (54.76%)	25 (83.33%)
PLR > 200	35 (48.61%)	14 (33.33%)	21 (70%)
LMR > 3	36 (50%)	27 (64.28%)	9 (30%)

Results are presented as median and interquartile range. Median survival time is presented as median and 95% confidence intervals. NLR—neutrophil to lymphocyte ratio; PLR—platelet to lymphocyte ratio; LMR—lymphocyte to monocyte ratio; WBC—white blood cells; CRP—C-reactive protein (available only for 13/40 patients in the resection group and for 7/30 patients in the palliative group); na—not applicable. ^a^ Presented as mean and standard deviation. ^b^ Presented as the number of cases and percentages. ^c^ N stage was available only for 42 subjects with resection. ^d^ Tumor–node–metastasis stage was available only for 54 subjects with resection or metastasis.

**Table 2:** Univariate and multivariate Cox proportional hazard regressions on subjects with Klatskin tumor adjusted for age, procedure, resection margin, and TMN stage.

	HR Unadjusted	(95% CI)	*p*	HR Adjusted *	(95% CI)	*p*	HR Adjusted ^#^	(95% CI)	*p*
NLR	1.13	(1.07–1.19)	<0.001	1.09	(1.02–1.15)	0.0057	1.07	(0.8032–1.427)	0.6422
PLR	1.004	(1.002–1.006)	<0.001	1.003	(1.001–1.005)	0.0017	1.002	(0.9981–1.007)	0.2726
LMR	0.84	(0.69–1.014)	0.069	0.9	(0.75–1.09)	0.31	1.26	(0.9945–1.609)	0.0555
SII	1.0003	(1.0002–1.0004)	<0.001	1.0002	(1.0001–1.0003)	0.0037	1.000	(0.9998–1.001)	0.1577
NLR ≥ median	2.39	(1.38–4.13)	0.0018	2.01	(1.06–3.83)	0.03	1.05	(0.4410–2.480)	0.9189
PLR ≥ median	2.02	(1.18–3.48)	0.0109	1.33	(0.73–2.4)	0.35	0.89	(0.3518–2.245)	0.8029
LMR ≥ median	0.54	(0.32–0.92)	0.024	0.7	(0.39–1.26)	0.24	1.79	(0.7201–4.434)	0.2107
SII ≥ median	2.29	(1.31–4.02)	0.004	1.64	(0.88–3.02)	0.12	1.22	(0.5012–2.953)	0.6648
NLR ≥ 3 (Yes vs. No)	1.8	(0.97–3.32)	0.0587	1.82	(0.94–3.55)	0.08	0.73	(0.3334–1.590)	0.4261
NLR ≥ 6 (Yes vs. No)	3.45	(1.81–6.58)	<0.001	2.85	(1.426–5.68)	0.003	2.19	(0.5081–9.478)	0.2924
PLR ≥ 150 (Yes vs. No)	1.98	(1.09–3.57)	0.024	1.54	(0.82–2.89)	0.17	0.79	(0.3208–1.980)	0.6250
PLR ≥ 200 (Yes vs. No)	1.89	(1.11–3.26)	0.02	1.238	(0.68–2.23)	0.49	0.89	(0.3518–2.245)	0.8029
LMR ≥ 3 (Yes vs. No)	0.53	(0.32–0.92)	0.024	0.71	(0.39–1.26)	0.24	1.79	(0.7201–4.434)	0.2107
SII ≥ 1200 (Yes vs. No)	2.1	(1.21–3.65)	0.007	1.73	(0.93–3.23)	0.08	0.96	(0.39–2.34)	0.93

HR—hazard ratio; CI—confidence interval; NLR—neutrophil to lymphocyte ratio; PLR—platelet to lymphocyte ratio; LMR—lymphocyte to monocyte ratio; SII—systemic immune-inflammation index. * Adjusted for age ≥ 65 years, procedure, T stage, M stage (for all subjects). ^#^ Adjusted for age ≥ 65 years, resection margin, and TNM stage (only for 42 subjects with resection).

## Data Availability

Not applicable.
